# Pseudotumor following total hip arthroplasty: experience of a tertiary referral center and proposal of the new “PCS” classification system

**DOI:** 10.1007/s00402-025-05766-3

**Published:** 2025-02-19

**Authors:** Davide Stimolo, Francesco Muratori, Lorenzo Cucurullo, Guido Scoccianti, Matteo Innocenti, Domenico Andrea Campanacci

**Affiliations:** 1 University of Florence, Florence, Italy; 2 Careggi University Hospital, Florence, Italy

**Keywords:** Pseudotumor, Total hip arthroplasty, Classification, PCS, Revision surgery, MoM

## Abstract

**Introduction:**

This study summarizes outcomes in treating pseudotumors of the hip at a tertiary referral center and introduces a classification system to aid treatment decisions and enhance communication among providers.

**Materials and methods:**

We collected data from 39 patients who underwent surgery for hip pseudotumor, analyzing implant failures based on patient history, revision reasons, bearing surface type, mass location and size, bone loss, revision type, and whether it was single- or two-stage. We introduce the PCS classification: ‘P’ for Pseudotumor (with ‘s’ for symptomatic, ‘e/I’ for intra/extrapelvic location, and ‘m’ for high Chromium/Cobalt levels), ‘C’ for implant status, and ‘S’ for bone loss extent. In 37 patients, we evaluated Cohen’s kappa coefficient to evaluate interobserver reliability.

**Results:**

Twenty (51.2%) patients were female, with a mean age of 71 years (range 36–89; σ 12.11); the mean follow-up duration was 54.43 months (range 12.2–128.3). The average size of the pseudotumor was 13.10 cm (range 3.3–37.2; σ 7.11) with 61.5% exhibiting extra-pelvic localization only. Bearing surfaces were MoM in 27 patients (69.2%). Single-stage revision surgery was performed in 87.1% of patients. There were 7 (17.9%) implant failures. No significant differences in failure rates were observed based on considered parameters. The agreement following Cohen’s coefficient for the combined PCS classification was k = 0.43. Moderate to almost perfect agreement was obtained for parameter P and S, with k = 0.48 for parameter C.

**Conclusion:**

No correlation was found between failures and analyzed characteristics. Our classification assesses clinical scenarios and stratifies surgical complexity for indication purposes. While interobserver agreement varies with parameter C, it is consistent with parameters P and S.

## Introduction

**Fig. 1:**
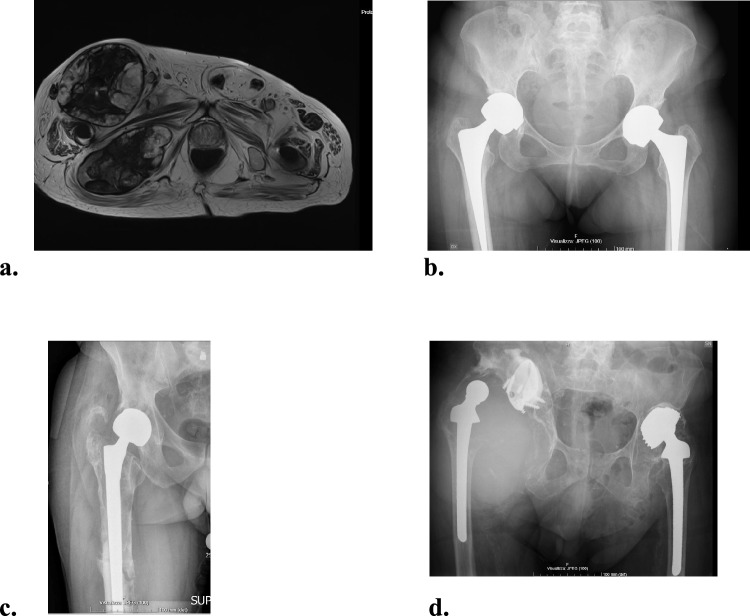
**a–d** MRI T2-weighted axial view reveals a large intra- and extrapelvic pseudotumor, classified as Anderson Grade C2, Matthies Class III, and Hauptfleisch Type III. These classification systems do not address implant status. Images **b**–**d** highlight potential scenarios linked to the pseudotumor, each posing unique technical challenges and reconstruction requirements

Pseudotumor after Total Hip Arthroplasty (THA) is defined as a non neoplastic nor infective solid or cystic neoformation characterized by a localized fibrous/granulomatous exudate and accumulation of liquids that develops around the hip replacement [1]. The main cause is believed to be a reaction to wear debris [2, 3], more common in Metal-on-Metal (MoM) or Metal-on-Polyethylene bearings (MoP) [4–7]. The inflammatory reactions can cause massive osteolysis and soft tissue disruption. The true incidence is not known and ranges from 0.5 to 4.6% [8, 9]. However, it is underestimated because many patients have asymptomatic pseudotumor in stable implants [10–13]. Current classification systems consider the radiological characteristics of masses on MRI but do not provide information about implant status or guide the type of surgery required (Fig. 1) [12, 14–17]. In addition, it is difficult to obtain significant results in studies because of the rarity and clinical variability of the pathology [8, 18, 19]. Thus, there are no approved treatment guidelines.

As our department is dedicated to Musculoskeletal tumors, patients with a periprosthetic mass are often referred to our center for the diagnostic pathway. The aim of the study was reporting our results in treatment of pseudotumor after THA and to propose a classification system with scientific, communicative, and therapeutic finalities.

## Material and methods

This is a monocentric retrospective study. We collected all cases of THA revision with a confirmed diagnosis of pseudotumor treated at our University Hospital in the period 2009–2023. Diagnosis of pseudotumor was confirmed by histologic report. Indications for revision surgery in the presence of a pseudotumor were given for one of the following reasons: mass-related (compression symptoms, elevated chromium and cobalt ions in the blood, mass volume) or implant-related (loosening, infection, instability, etc.). Periprosthetic Joint Infections were always ruled out before revision surgery. Patient consent was collected pre-operatively after they were informed of the procedure following the principles of the Declaration of Helsinki. Local Ethics Committees authorized the study with the following reference number: 27119_oss.

For every patient, we recorded: anamnestic data, cause of revision, tribology, mass localization (extrapelvic, intrapelvic, both intra and extrapelvic), mass dimensions, acetabular and femoral bone loss according to Paprosky classification [20, 21], type of revision (partial, total), complications after revision surgery and implant failure rates. The mass dimension was measured on CT scan or MRI with metal artifact reduction protocols, and we recorded the major diameter. We considered as failures any adverse event that required surgery and component exchange (partial or total). We compared failure rates based on the following parameters: mass characteristics, grade of bone loss, surgery received, number of previous surgeries, bearing. We compared failure rates based on the parameters described earlier. In cases requiring two-stage revision, we compared bone loss, mass characteristics, and the necessity of custom-made implants with the rest of the population.

We included patients with at least one year of follow-up (FU). We excluded patients with a diagnosis of pseudotumor of other joints, patients affected by pseudotumor who were not treated surgically, patients with incomplete data, and bilateral procedures (to reduce the risk of bias arising from complications caused by repeated surgical procedures).

### PCS classification system

Our second aim was to propose the PCS classification system: P = Pseudotumor; C = Cup status; S = Stem status (Table 1). It takes inspiration from the TNM classification system for malignant tumors [22] and includes clinical, radiological, and laboratory parameters, and describes the implant’s status.

**Table 1:** PCS classification system

Pseudotumor (P)	a/s	Asymptomatic/Symptomatic
	e/i	Extrapelvic only/Intrapelvic component
	m	Metal ions blood level > 7 mg/L
Cup (C)	1	Stable with minor bone loss
	2	Loose with minor bone loss
	3	Loose with severe bone loss
	x	Not able to define the entity of bone loss or the status of cup stability
Stem (S)	1	Stable with minor bone loss
	2	Loose with minor bone loss
	3	Loose with severe bone loss
	x	Not able to define the entity of bone loss or the status of stem stability

Normal Cr level ≤ 5 mcg/L, Normal Co level ≤ 2 mcg/L. Governmental agencies, such as the Medicines and Health Care Products Regulatory Agency in the United Kingdom, have stated that serum Cr and Co metal ion levels > 7 mcg/L or Cobalt > 10 mcg/L are worrisome in the setting of unilateral MOM THA[23]

Cup: Minor bone loss Paprosky I-IIA, Severe bone loss Paprosky IIB-IIIB

Stem: Minor bone loss Paprosky I-II, Severe bone loss Paprosky IIIA-IIIB

The term P describes pseudotumor characteristics. It is followed by qualifications. Qualification “a” or “s” indicates the presence of clinical symptoms. For symptoms, we include pain, reduced function, swelling, instability, and compression symptoms. Qualification “e” (extrapelvic) or “i" (with intrapelvic involvement) refers to the mass localization. The qualification “m” represents elevated metal ions blood levels (Chromium, Cobalt or both). The qualification “m” can be omitted if negative. In the best situation the mass is asymptomatic and entirely extrapelvic “Pae”. In the worst case, the mass is symptomatic, with intrapelvic involvement and elevated metal ions “Psim”.

The terms C and S identify the implant status. They are followed by a number representing increasing complexity for reconstruction. The stability of the implants can be evaluated on plain hip and pelvis x-rays and/or on CT scan. In cases where it is not possible to define loosening or bone loss the qualification “x” should be used.

**Fig. 2:**
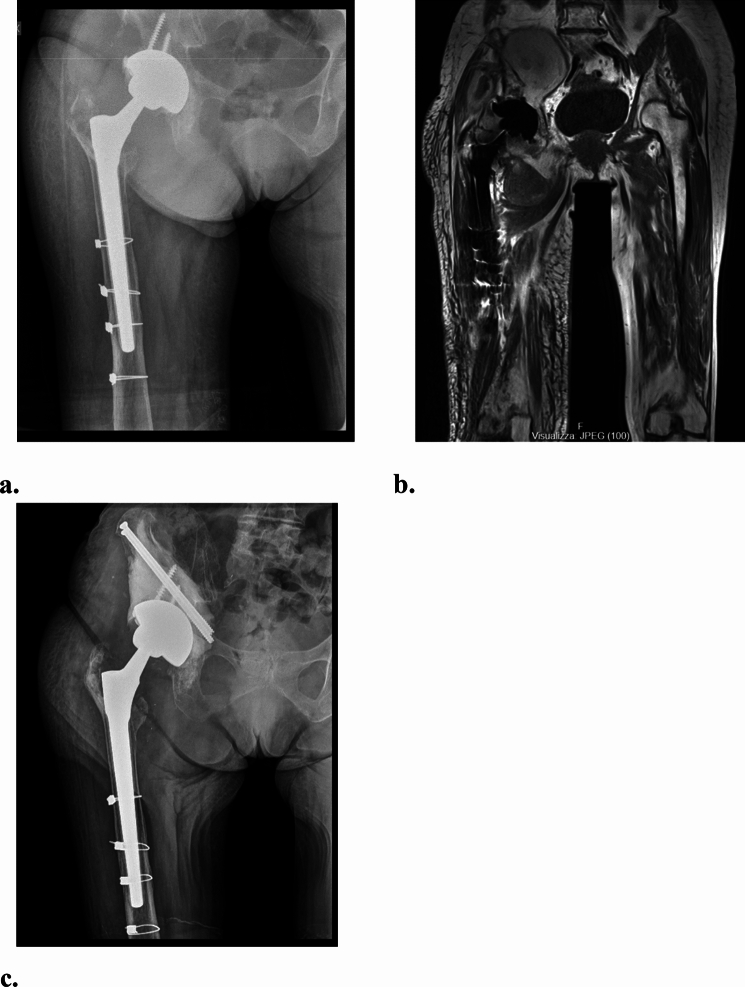
**a–c** An 84-year-old female presented with soft tissue swelling and pain, showing a large intrapelvic pseudotumor and normal blood metal ion levels. The stable Metal-on-Polyethylene cup and stem classified the case as Psi C1 S1 (PCS). Treatment involved preoperative embolization, mass debulking, and replacing the head and liner with ceramic and polyethylene. Despite an ischial bone defect, cup stability remained intact. The defect was filled with cement and secured with screws. **a** Preoperative anteroposterior view of the right hip; **b** T1w-MARS MRI, coronal view showing large intrapelvic and extrapelvic lesions; **c** Postoperative anteroposterior x-ray of the right hip

**Fig. 3:**
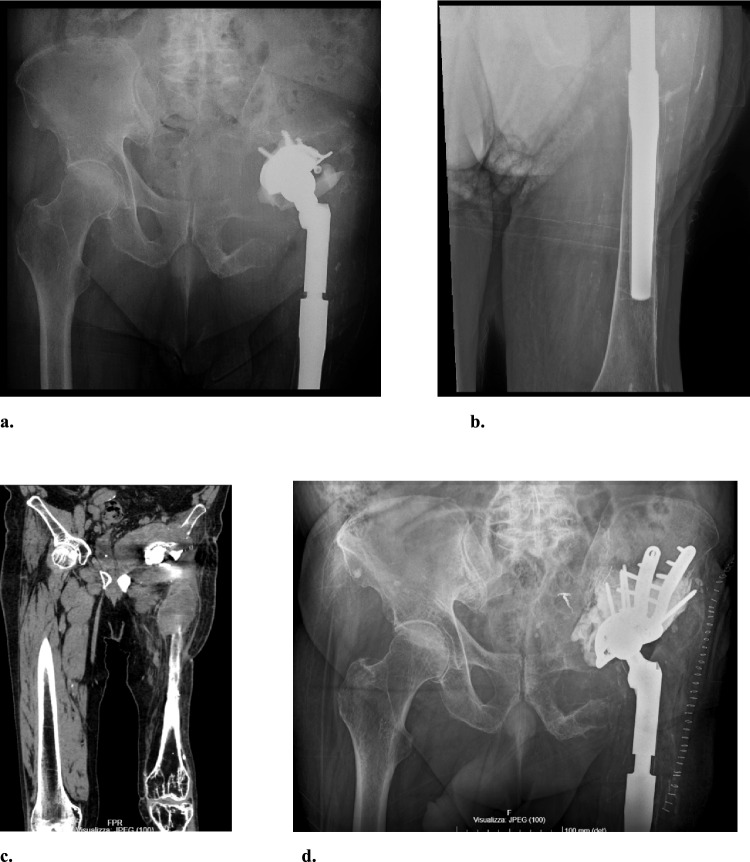
**a–d** A 76-year-old male presented with a painful left limb mass. Coronal CT showed a large pseudotumor in intra- and extrapelvic regions with extensive osteolysis. The cup was loose (Paprosky 3B), but the femoral stem remained stable. Metal-on-Polyethylene bearings were used, with negative metal ion levels. The PCS classification categorized the case as Psi C3 S1. Treatment included preoperative embolization, mass debulking, cup revision with a cage and cemented dual mobility cup, and head exchange. **a** Preoperative anteroposterior pelvic x-ray; **b** Preoperative anteroposterior x-ray of the left thigh, focusing on the distal stem tip; **c** CT scan OMAR coronal view showing a large pseudotumor with severe osteolysis; **d** Postoperative anteroposterior pelvic x-ray

Every indicator should be separately evaluated preoperatively and expressed in capital letters, followed by qualification (term P) or number (C and S). The use of the classification system is illustrated in Figs. 1 and 2 through clinical cases (Figs. 2, 3).

We applied the PCS classification to a cohort of 37 patients. We included all patients treated at our center between 2009 and 2023, even those with a follow-up shorter than one year or bilateral procedures. We excluded patients without all necessary X-rays, MRI, or CT scans available for multiple reviews. Interrater reliability between the two investigators (DS and FM) was calculated using Cohen’s kappa, and it was reported along with the 95% confidence intervals (CI). Initially, the variability was assessed for the combined PCS parameters, and then for each parameter individually (P, C, and S) to determine if one influenced the reliability of the classification system more than the others.

### Data analysis

All statistical analyses were performed using SPSS software version 21 (SPSS Inc., Chicago, IL, USA). The Kolmogorov–Smirnov test was used to check the normal distribution of the continuous variables and thus the t-test was used for unpaired and paired continuous variables, and the chi-square test was applied for categorical variables. A multivariate logistic regression model was also performed to estimate the odds ratio (OR) of treatment failure including patients’ Paprosky Classification of Acetabular Bone Loss, Paprosky Classification of Femoral Bone Loss and maximum Pseudotumor dimension as covariates. Statistical significance was set at p < 0.05.

The reliability for combined PCS and for P parameters was assessed by calculating an unweighted Cohen’s kappa, whereas the agreement for C and S parameters was assessed providing unweighted and weighted Cohen’s kappa estimates, applying a linear weighting for the latter. For both unweighted and weighted kappas, reliability was classified based on its values as: None (0–0.20), Minimal (0.21–0.39), Weak (0.40–0.59), Moderate (0.60–0.79), Strong (0.80–0.90) and Almost Perfect (> 0.90), as reported [24].

## Results

### Population data

We collected 43 cases of THA revision and associated pseudotumor. Three patients were excluded because of bilateral surgery, one was excluded due to a follow-up shorter than 12 months. Finally, we included 39 patients, 19 (48.8%) male and 20 (51.2%) female. The mean age at revision surgery was 71 years old (range 36–89; σ 12.11). Mean BMI was 24.38 (range 17.78–31.22; σ 3.581). The mean follow-up was 54.43 months (range 12.2–128.3 months). 89.7% of patients were symptomatic, and the main cause of revision was aseptic loosening (21/39 patients, 53.8%) (Table 2).

**Table 2:** Causes of revision arthroplasty

Causes of revision	
Aseptic loosening	21–53,8%
Instability/dislocations	7–17,9%
Soft tissue related pain	5–12,8%
Periprosthetic fracture	2–5,1%
Elevated ions level	2–5,1%
Periprosthetic joint infection	2–5,1%

The mean dimension of the pseudotumor was 13.10 cm (range 3.3–37.2; σ 7.11). The localization was entirely extrapelvic (Ex) in 24 patients (61.5%), entirely intrapelvic (In) in 3 (7.7%) and combined Ex + In in 12 patients (30.8%). The acetabular bone loss was minor, grade I-IIA, in 20 patients (51.3%) and severe, grade IIB-IIIB, in 19 patients (48.7%); the femoral bone loss was minor, grade I-II, in 29 patients (74.3%) and severe, grade III, in 10 patients (25.6%). The bearing was MoM in 27 patients (69.2%), MoP in 9 patients (23.1%), CoP in 2 patients (5.1%) and CoC in 1 patient (2.6%). There was no difference in intrapelvic involvement (p = 0.102), mean dimension (p = 0.139), severity of bone defect based on bearing surfaces.

Revision surgery was performed in a single stage in 34 patients (87.1%) while two-stage mode was adopted for 5 patients (12.8%). Details are summarized in Table 3. We implanted a total of 4 custom-made prosthesis, two in the two-stage revision group, and two in the other group of patients. The difference in use of custom-made implants was not statistically significant (p = 0.065).

**Table 3:** Revision surgery for pseudotumor, details

One-stage	34 (87.1%)
Two-stage	5 (12.8%)
Total revision	10 (25.6%)
Partial revision	27 (69.2%
Pseudotumor excision	2 (5.1%)
Partial revision	
Polyethylene & Head Exchange	4 (14.9%)
Cup revision	18 (66.7%)
Stem revision	5 (18.5%)
Implants	
Cups	27
Trabecular metal	14 (51.8%)
Cage/cup cage	9 (33.3%)
Custom made	4 (14.9%)
Stems	14
Non-modular revision stem	3 (21.4%)
Modular revision stem	5 (35.7%)
Proximal femur replacement prosthesis	6 (42.8%)

We had 7 (17.9%) implant failure on a total of 10 reoperations (25.6%) (Table 4). We defined failures among reoperations as cases requiring a partial or total revision of the implant. Table 5 represents the complication and failure rates based on population characteristics. We did not observe a difference in complication and failure rates based on dimensions, localization, tribology and severity of bone loss. The results of multivariate logistic regression suggest that femoral and acetabular bone loss and dimension of the pseudotumor are not significantly associated to the risk of treatment failure (Table 6).

**Table 4:** Description of complications after revision surgery for pseudotumor; in brackets number of implant failures. The percentage refers to the entire population (39 patients). Single episodes of dislocations without evident implant malpositioning were treated conservatively. One patient with loosening refused revision surgery. PJI was acute and resolved with DAIR. Wound healing problems solved with dedicated professional nurse management. Both periprosthetic fractures required surgery but one underwent open reduction and internal fixation as the implant was stable

Complications		
Instability/dislocation	6 (3)	15,4%
Aseptic loosening	4 (3)	10,2%
Acute PJI	1	2,6%
Wound healing related	4	10,2%
Periprosthetic fracture	2 (1)	5,1%
Medical complication	2	5,1%
Residual pain and swelling	1	2,6%

*PJI* periprosthetic joint infection

**Table 5:** Failure rates based on population characteristics. Minor acetabular bone loss Paprosky grade I-IIA. Severe acetabular bone loss Paprosky grade IIB-IIIB. Minor femoral bone loss Paprosky grade I-II. Severe femoral bone loss Paprosky grade III

Data	Total on population	Failures
Sex F (n,%)	20 (51.2)	3 (15)
Sex M (n,%)	19 (48.7)	4 (21)
Age (mean)	70.7	62.6
BMI	24.38	24.52
Pseudotumor dimension (mean cm)	13.10	10.7
First revision (n,%)	21 (53.8)	4 (19)
Multiple revisions (n,%)	18 (46.1)	3 (16.7)
Intrapelvic mass (n,%)	14 (35.9)	3 (21.4)
Extrapelvic only (n,%)	25 (64.1)	4 (16)
MoM (n,%)	27 (69.2)	5 (18.5)
MoP (n,%)	9 (23)	2 (22.2)
Minor acetabular bone loss (n,%)	20 (51.2)	4 (29)
Severe acetabular bone loss (n,%)	19 (48.7)	3 (15.8)
Pelvic discontinuity (n,%)	10 (25.6)	3 (33.3)
Minor femoral bone loss	29 (74.3)	6 (20.7)
Severe femoral bone loss	10 (25.6)	1 (10)

**Table 6:** Results of the multivariate logistic model to estimate risk of treatment failure

	OR	95% CI	p-value
Paprosky femoral minor	Ref	–	
Paprosky femoral major	1.46	0.27–7.93	0.66
Paprosky acetabular minor	Ref	–	
Paprosky acetabular major	0.49	0.05–5.20	0.55
Maximum pseudotumor dimension (cm)	0.94	0.81–1.10	0.45

### PCS Classification

**Fig. 4:**
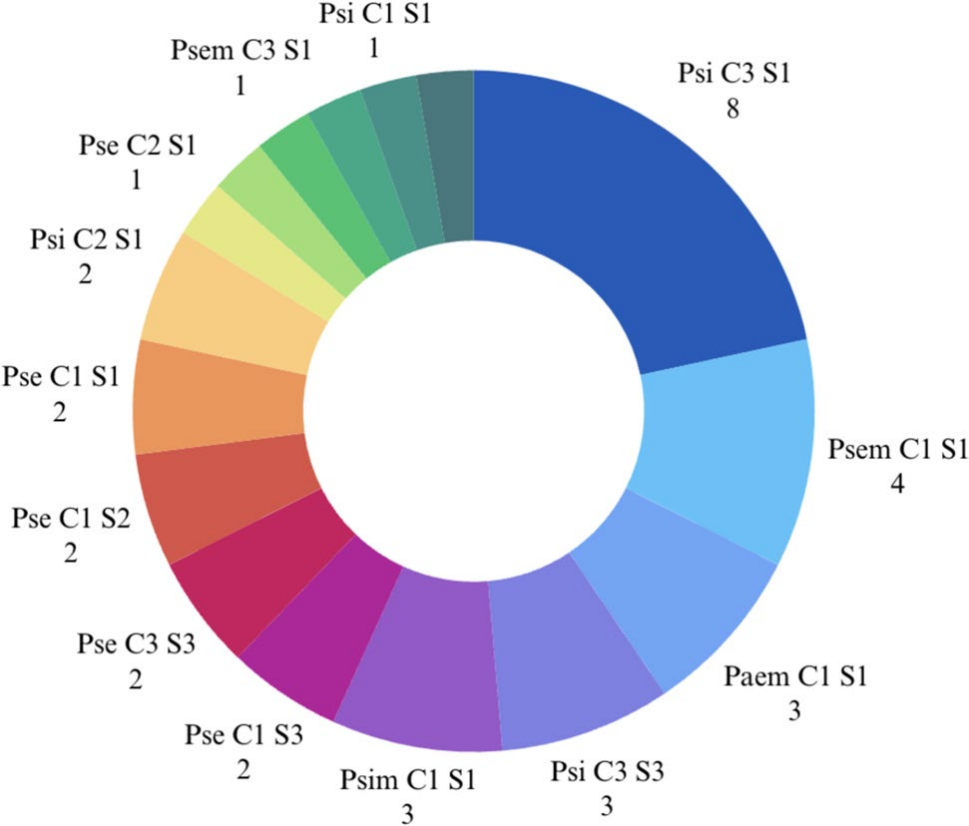
Distribution of PCS Phenotypes

Phenotypes based on classification are reported in Fig. 4.

Results on interobserver variability showed weak agreement for the combined unweighted PCS (Cohen’s k = 0.43, 95% CI 0.26–0.59), moderate to strong agreement for the P and S parameters, and weak agreement for the C parameter (Table 7).

**Table 7:** Cohen’s kappa values for the combined PCS classification and each parameter, with parameter P assessed for different qualifiers. CI Confidence interval

Parameter	Cohen’s k unweighted	CI 95%	Agreement level
PCS	0.43	0.26–0.59	Weak
Pa/s	0.64	0.19–1	Moderate
Pe/i	0.84	0.66–1	Strong
Pm	0.94	0.83–1	Almost perfect
C	0.48	0.27–0.69	Weak
S	0.70	0.46–0.93	Moderate

Therefore, the “weak” agreement observed in the combined PCS is likely due, for the most part, to the weak agreement concerning the Cup parameter.

## Discussion

### Results

We found no difference in failure rates based on patient history, mass characteristics, tribology, or bone loss severity. With the numbers available, we could only demonstrate younger patients appeared at higher risk of failure, possibly due to greater functional demands and longer time to develop complications.

Most studies have limited patient numbers, reducing the likelihood of significant findings [1, 2, 5, 9, 11, 25, 26]. With 39 patients, our study is robust. We observed a 17.9% failure rate, primarily due to dislocation. This aligns with recent literature, which reports reoperation rates of 10–25% after ARMD revisions [27–30] —higher than the 14% rate seen in revisions for other causes [25]. Dislocation is the most common complication across studies, with solid masses and severe abductor damage linked to higher risk. In our series, first-time dislocations were treated conservatively with closed reduction and a hip brace, while recurrent dislocations were managed surgically. A constrained liner was required in two out of three cases.

Another reason for the lack of significant results could be the inclusion of a heterogeneous population without accounting for preoperative comorbidities. We recognized these limitations but aimed to identify surgical-related causes of failure to correlate with classification parameters. However, our findings confirm that the reconstructive strategy can follow the traditional hip revision algorithm [20, 21] and that complete excision of the pseudotumor is not necessary once the source of debris has been removed [6, 31, 32]. We fully excised extrapelvic masses and partially debulked accessible intrapelvic extensions. Literature shows mixed results: Almousa et al. [33] found only 1 recurrence in 5 cases with limited debulking, while Liddle [34] suggested incomplete excision could lead to early recurrence. Grammatopoulos et al. [25] observed recurrences in 3 of 5 cases, attributing it to remaining wear debris. Liow [30] reported taper corrosion in recurrence cases, while recent case reports note recurrences treated by aspiration or further revision due to severe wear [35, 36].

When dealing with large pseudotumors or complex joint reconstruction a two-stage procedure represents a valid option [37]. The goal of custom-made reconstruction might also warrant consideration for a two-stage procedure because the absence of metal artifacts on CT scan allows for more precise planning. Indeed, we tended to use more custom-made cup implants in the two-stage revisions (p = 0.065).

### Existing classification systems

The pseudotumor classifications by Anderson [15], Matthies [38], and Hauptfleish [14] rely on MRI findings, while Boomsma’s classification is CT-based and not specific to pseudotumor evaluation [17]. MRI grading systems link an increase in solid components to a higher risk of symptoms, helping to identify patients at greater risk for revision surgery [9, 12, 39, 40]. Boomsma recommends revision surgery for patients in Class B and C. Van der Weegen suggested treating hip resurfacing MoM based on Anderson’s classification, patient symptoms, and cobalt and chromium blood levels, with follow-up for C1-C2 pseudotumors, cobalt and chromium levels below 7 ppb, and asymptomatic patients [10]. Limitations of these classifications are that they require a radiologist experienced in MoM disease and that they do not provide information about the implant status.

### PCS classification

The PCS classification system integrates key parameters essential for guiding indications for revision surgery and determining the necessary surgical treatment. The classification functions like a flowchart, requiring a step-by-step assessment of the patient and reconstructing a logical evaluation pathway. Additionally, it serves as a checklist; if the provider has not analyzed all parameters, they cannot classify the patient. It facilitates communication and aids in surgical planning, and is applicable regardless of the prosthetic bearing. The system is based on the widely recognized TNM staging system and incorporates the globally utilized Paprosky classification, facilitating memorization and implementation in the clinical practice. This classification uses readily available and reproducible parameters, aiding in the objective selection of candidates and helping to identify potential pitfalls, complications, and complexities of the procedure.

To describe the pseudotumor we decided to use qualifiers: symptoms, localization, and Co and Cr ions. The presence of symptoms is significant because it is often associated to high-grade pseudotumors. Additionally, symptomatic patients typically warrant a lower threshold for surgical intervention. Mass localization serves as an index for assessing the difficulty of excision, determining potential contiguity with vital structures, evaluating the necessity of an endopelvic approach, and considering the need for vascular surgeon support or preoperative embolization. Elevated metal ion levels serve as an indicator of potential ARMD. Even in asymptomatic patients, these levels can escalate to toxic levels, necessitating revision surgery. We needed to explicitly mention the qualification because many patients with pseudotumors exhibit negative ion levels [10, 41]. Regarding the implants, it is important to recognize component loosening and to detect the extension of bone loss. Accordingly, bearing exchange can be done with partial or total revision and adequate surgical strategy can be planned. In cases of massive pelvic or/and femoral bone loss (any P C3 S3) two-stage surgery may be the optimal approach.

The classification encompasses most of the possible combinations and scenarios, ranging from patients suitable to FU to patients with massive femoral and acetabular bone loss in which we can consider a two-stage procedure.

PCS Classification can potentially help to identify candidates for follow-up and patients who require close monitoring to address issues before massive bone loss develops, enabling more conservative revision surgery. For patients with stable implants and minor or no bone loss (C1 and S1), the decision to proceed with follow-up or revision surgery depends on the parameter "P."

In asymptomatic patients with metal ion blood levels under the normal cutoff (Pae C1 S1), it is possible to recommend an initial follow-up at six months. If the situation remains unchanged, an annual follow-up with x-rays, MRI, metal ion testing, and clinical examination is advised.

If the patient is symptomatic but metal ion levels are negative or slightly positive (Pse C1 S1), strict follow-up with x-rays and metal ion testing every 3–6 months is necessary. Surgery should be considered at a low threshold if symptoms worsen or metal ion levels rise in the blood.

In cases of intrapelvic extension of the mass, physicians should carefully evaluate and investigate potential compression symptoms affecting pelvic organs and neural roots.

### Interobserver validation

Even though the combined interobserver variability for the PCS classification (k = 0.43) was weak, it performed better than the Matthies classification (k = 0.23) and the Hauptfleisch classification (k = 0.34). The Anderson classification is associated with a higher Cohen’s kappa coefficient (k = 0.58), but its reliability is still considered weak [42, 43]. However, if we consider that the other classifications focus only on pseudotumor characteristics, our P parameter and its subqualifications allow the PCS classification to outperform the others (weak vs moderate to almost perfect agreement). Moreover, while other classifications provide prognostic value and must be performed by experienced radiologists, the PCS classification can be easily utilized by orthopaedic surgeons. The weak agreement observed in the combined PCS classification is primarily due to the Cup parameter. This may stem from using a simplified Paprosky classification, which was included because it is the most widely used system for describing acetabular bone loss in revision surgery. Unfortunately, reliably evaluating the cup remains a significant challenge in hip revision surgery. A recent systematic review [44] comparing the reliability of the Paprosky, American Academy, Gross, and Saleh classifications demonstrated low reliability across these systems, particularly noting a Cohen’s kappa of 0.45 (weak) for the Paprosky classification. In the study by Driscoll [45], poor to weak agreement was observed in the use of the Paprosky classification, regardless of the examiner’s experience, and there was also poor agreement between classification and treatment performed.

## Limitations

Our series includes only patients treated surgically, excluding results in patients who could be managed with conservative treatment. Our hypothesis is that our classification can be also used to individuate patients candidate to FU. ALVAL is associated with two histological phenotypes, macrophage dominant and lymphocytes predominant [46]. However, the clinical implications of this association remain unclear, and therefore, we did not incorporate it into the classification. The strength of the study lies in its follow up period, with up to 41% of patients having a FU longer than 5 years, which provides a reliable timeframe to analyze the results of revision surgery.

## Conclusion

Complications and failures are common after revision hip arthroplasty for pseudotumors, often due to instability. Complete removal of the pseudotumor may not always be necessary, particularly for those intrapelvic, once the source of wear debris is eliminated. Two-stage revision may be beneficial for suspected infection or custom-made reconstruction. We introduce the novel “PCS classification” system to evaluate clinical scenarios, stratify surgical complexity, and document crucial parameters for surgical indication and potential prognostic factors.

Interobserver agreement is affected by parameter C, but it performs well when considering parameters P and S. However, the classification does not provide prognostic results for revision surgery due to limited data.

## Data Availability

No datasets were generated or analysed during the current study.
